# Comparing the representation of medicinal products in RxNorm and SNOMED CT – Consequences on interoperability

**Published:** 2019-08

**Authors:** Jean Noel Nikiema, Olivier Bodenreider

**Affiliations:** a Bordeaux Population Health Research Center, ERIAS, Univ. Bordeaux, Inserm UMR 1219, F-33000, Bordeaux, France; b U.S. National Library of Medicine National Institutes of Health Bethesda, Maryland, USA

**Keywords:** RxNorm, SNOMED CT, medicinal products

## Abstract

**Objectives:**

To compare the representation of medicinal products in RxNorm and SNOMED CT and assess the consequences on interoperability.

**Methods:**

To compare the two models, we manually establish equivalences between the types and definitional features of medicinal products entities in RxNorm and SNOMED CT. We highlight their similarities and differences.

**Results:**

Both models share major definitional features including ingredient (or substance), strength and dose form. SNOMED CT is more rigorous and better aligned with international standards. In contrast, RxNorm contains implicit knowledge, simplifications and ambiguities, but its model is simpler.

**Conclusions:**

Since their models are largely compatible, medicinal products from RxNorm and SNOMED CT are expected to be interoperable. However, specific aspects of the alignment between the two models require particular attention.

## Background

Drug terminologies, such as RxNorm and the medicinal product hierarchy of SNOMED CT (Systematized Nomenclature of Medicine-Clinical Terms), support multiple use cases, including electronic prescription, drug information exchange, medication reconciliation, and analytics (including pharmacovigilance) (1,2). A formal representation of medicinal products is needed for the principled development and maintenance of such drug terminologies, as well as for precisely aligning existing drug terminologies (3).

Many definitional characteristics of medicinal products are similar among drug terminologies. For example, clinical drugs are generally defined in terms of ingredient, strength and dose form. However, the level of formality and the formalism used for representing medicinal products may differ among terminologies. Some attributes may also be specific to some terminologies (especially for country-dependent attributes, such as packaging information).

In addition to existing drug terminologies, international standards have been developed for the representation of medicinal products, such as IDMP (Identification of Medicinal Products). IDMP (4), a collection of recommendations from the International Standards Organization (ISO).

Interoperability among drug terminologies is especially important for exchanging drug information internationally. For example, a medication list established with RxNorm in the U.S. could be made available to any electronic health record (EHR) system in the world, in which drugs are represented using SNOMED CT. To fully support this use case, however, the models of medicinal products in RxNorm and SNOMED CT must be compatible, such that one can be accurately translated into the other.

We focus on RxNorm and SNOMED CT, because RxNorm is the standard drug terminology in the U.S. and SNOMED CT is the largest clinical terminology in the world, supported by a consortium of over 40 countries. While the RxNorm model has been analyzed (5,6), and reused to create others standards (7,8) and to integrate drug terminologies worldwide (8), there has not been a detailed comparison between RxNorm and SNOMED CT. Moreover, the SNOMED CT model for medicinal products is particularly interesting, because it was recently updated, in part to comply with IDMP requirements (9).

In this investigation, we compare the representation of medicinal products in RxNorm and SNOMED CT. The objective of our work is to analyze their similarities and differences and the consequences of these differences on interoperability between the two terminologies.

## Methods and results

In this section, we describe the models of RxNorm and SNOMED CT with focus on their definitional characteristics. Then we identify similarities and differences between the two models.

### The SNOMED CT model for medicinal products

The SNOMED CT, the largest clinical terminology in the world, is an international clinical terminology based on a formal concept model (10). SNOMED CT recently published a new model for the representation of medicinal products integrating requirements from IDMP (9). The model was developed to support international usage. Therefore, it is restricted to generic drugs and does not represent packaging information or branded drugs, which tend to be country-specific.

In accordance with requirements from IDMP, clinical drugs are represented in a closed worldview. This means that characteristics used to define clinical drugs must be sufficient and what is not stated is false. In contrast, in the open worldview, what is not stated is potentially true. For example, the representation of a clinical drug containing Atorvastatin must clearly state that this product only contains the substance Atorvastatin as its active ingredient (i.e., without any other active ingredient). In the open worldview, products containing Atorvastatin could also contain other active ingredients, e.g., Amlodipine.

**Figure 1–:**
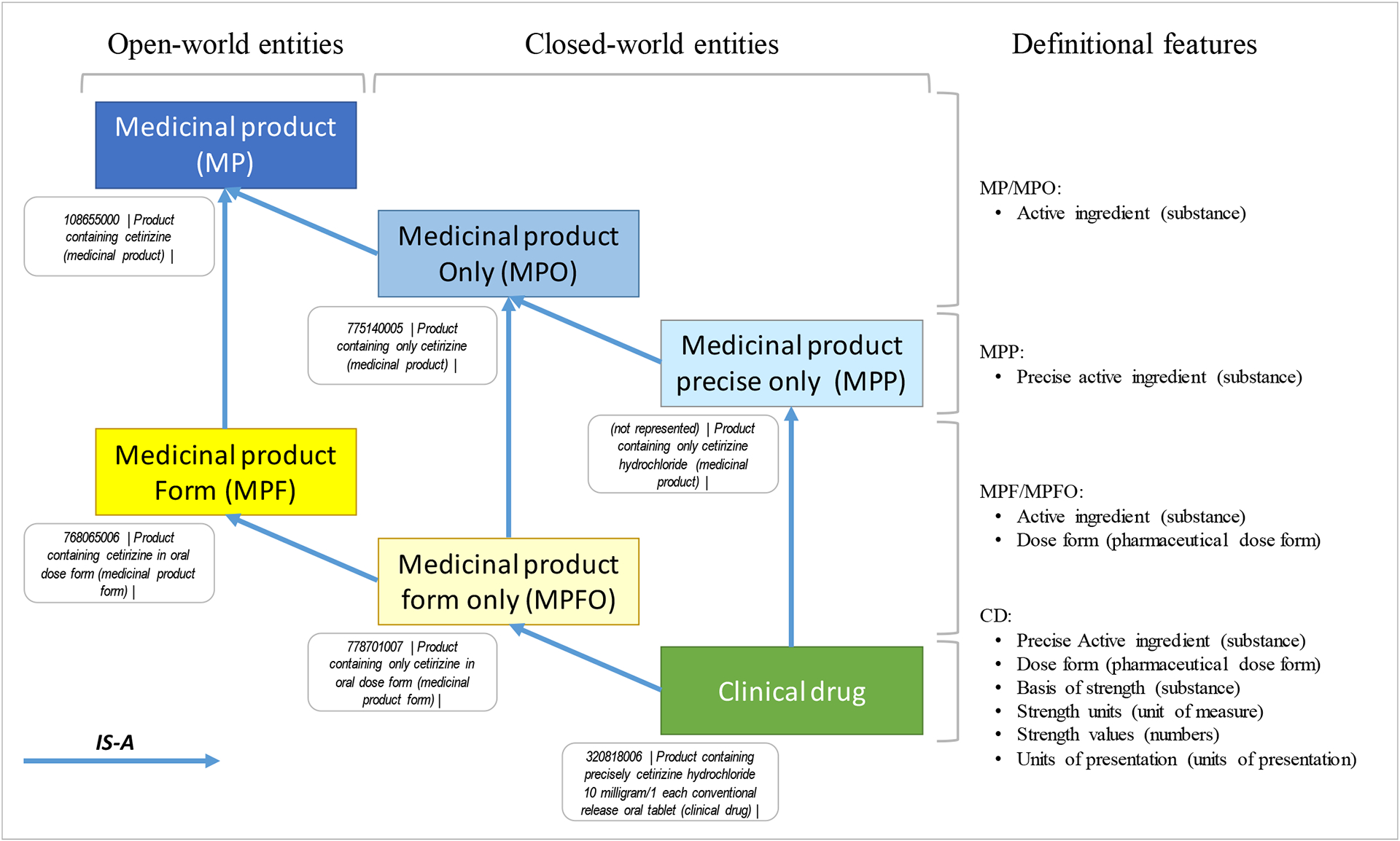
SNOMED CT model for the representation of medicinal products showing the six types of entities defined in the model, along with their definitional features and examples from the SNOMED CT terminology

As shown in Figure 1, the representation of medicinal products in SNOMED CT is based on a model with six (6) entities, arranged in a subclass hierarchy:

- Two ***medicinal product*** entities, in open and closed worldview (e.g., open worldview: *108655000* | *Product containing cetirizine (medicinal product)* and closed worldview: *775140005* | *Product containing only cetirizine (medicinal product)*).
- Two ***medicinal product form*** entities, in open and closed worldview, (e.g., open worldview: *768065006* | *Product containing cetirizine in oral dose form (medicinal product form)* and closed worldview: *778701007* | *Product containing only cetirizine in oral dose form (medicinal product form)*).
- One ***medicinal product precisely*** entity in closed worldview only (optional entity, currently not represented in SNOMED CT – hypothetical example: *Product containing only cetirizine hydrochloride (medicinal product)*).
- One ***clinical drug*** entity, in closed worldview only (e.g., *320818006* | *Product containing precisely cetirizine hydrochloride 10 milligram/1 each conventional release oral tablet (clinical drug)*).

The representation of SNOMED CT entities is based on “definitional roles” and related “types of values” in SNOMED CT (Figure 1):

- **Substance** is the type of values for the *active ingredient*, *precise active ingredient* and *basis of strength* roles, for example *372523007* | *Cetirizine (substance)* and *108656004* | *Cetirizine hydrochloride (substance)*. (The basis of strength is the substance in reference to which strength is defined.)
- **Unit of measure** is the type of values for the *strength unit* roles, for example, *258684004* | *milligram (qualifier value)*.
- **Number** is the type of values for the *strength value* roles, for example, *3445001* | *10 (qualifier value)*.
- **Pharmaceutical dose form** is the type of values for the *manufactured dose form* role, for example, *421026006* | *Conventional release oral tablet (dose form)*.
- **Unit of presentation** is the type of values for the *unit of presentation* role, for example, *732936001* | *Tablet (unit of presentation)*.

Closed-worldview are “closed” with respect to their active ingredient(s). More specifically, medicinal product and medicinal product form entities are closed with respect to their active ingredient(s), while medicinal product precisely and clinical drug entities are closed with respect to their precise active ingredient(s).

There are no hierarchical relations among substances. However, there is a “modification of” relation between a modified substance (e.g., ester or salt) and the corresponding base substance (e.g., between Atorvastatin calcium and Atorvastatin). Modified substances can be further modified.

IDMP requires that dose forms be defined in reference to a list of dose forms from the European Directorate for Quality in Medicines (EDQM). EDQM distinguishes between dose forms and units of presentation. Units of presentation are used to express the strength and quantity in countable entities, while dose forms correspond to the physical structure of the medicinal product.

In accordance with requirements from IDMP, strength units in SNOMED CT are aligned with the international standard for units of measure, UCUM (Unified Code for Units of Measure).

Finally, depending on the unit of presentation, strength can be represented as concentration strength, presentation strength or both.

### The RxNorm model

Created in 1992, RxNorm is a normalized terminology for clinical drugs in the U.S. RxNorm represents both generic drugs and branded drugs, as well as packs (11). The full model of RxNorm contains ten entities, five for generic drug entities and five for branded drugs entities. For comparison with SNOMED CT, we only present RxNorm generic drug entities and also omit packs.

**Figure 2–:**
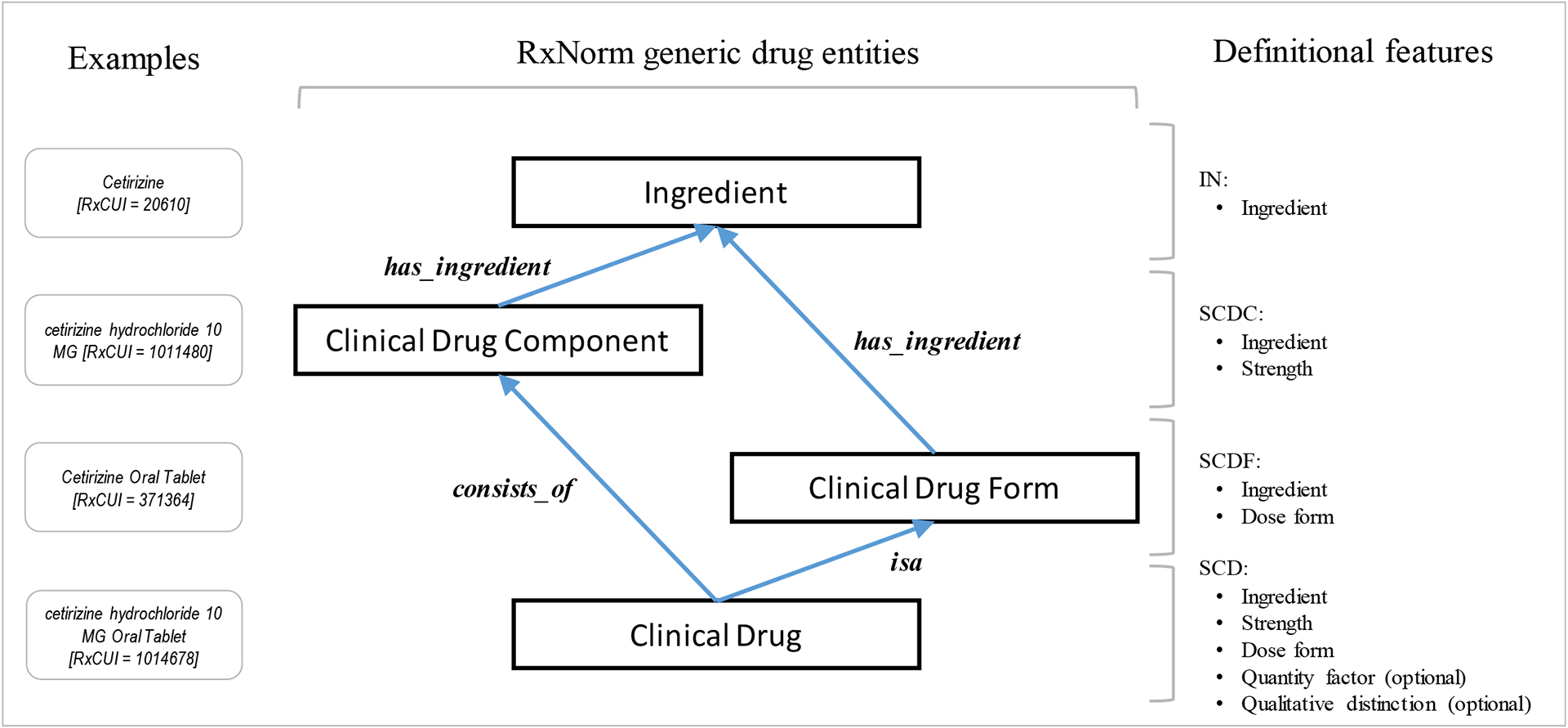
Simplified RxNorm model for the representation of generic medicinal products showing the four types of entities defined in the model, along with their definitional features and examples from the RxNorm terminology

The simplified RxNorm model for generic drug entities includes four entities (Figure 2):

- ***Ingredient***, including base ingredient (IN), precise ingredient (PIN), and multi-ingredient (MIN) (e.g., IN: *Cetirizine [RxCUI = 20610]*, PIN: *cetirizine hydrochloride [RxCUI = 203150]*, MIN: *Cetirizine / Pseudoephedrine [RxCUI = 352367]*)
- ***Clinical drugs component*** (SCDC), combining ingredient and strength (e.g., *cetirizine hydrochloride 10 MG [RxCUI = 1011480]*)
- ***Clinical drugs form*** (SCDF), combining ingredient and dose form (e.g., *Cetirizine Oral Tablet [RxCUI = 371364]*)
- ***Clinical drug*** (SCD), combining ingredient, strength and dose form (e.g., *cetirizine hydrochloride 10 MG Oral Tablet [RxCUI = 1014678]*)

The representation of these entities relies on three mandatory and two optional definitional features:

- Mandatory definitional features:
  - ingredient (IN/PIN/MIN) (e.g., IN: *Cetirizine [RxCUI = 20610]*, PIN: *cetirizine hydrochloride [RxCUI = 203150]*, MIN: *Cetirizine / Pseudoephedrine [RxCUI = 352367]*)
  - dose form (DF) (e.g., *Oral Tablet [RxCUI = 317541]*)
  - strength (e.g., *10 MG*)
- Optional definitional features (see below for examples):
  - quantity factor (QF)
  - qualitative distinction (QD)

Strength in RxNorm is normalized. In its units of measure (e.g., for volume, weight, surface), RxNorm uses one unit for each type quantity (e.g., milligram for weight rather than gram or microgram).

The representation of dose forms in RxNorm is not based on a specific standard (12). It is also important to note that the SCDs and SCDCs refer to the basis of strength substance (e.g., cetirizine hydrochloride), while SCDFs refer to the base ingredient (e.g., cetirizine). Of note, ingredients in RxNorm can (purposely) be understood as either the substance contained in a medicinal product as active ingredient (e.g., “cetirizine the substance”) or the class of all medicinal products containing this substance as active ingredient. Precise ingredients (PINs) generally correspond to modified forms of the corresponding base ingredients (INs). PINs cannot be further modified.

In addition, RxNorm does not explicitly have a notion of “worldview” (i.e., open or closed worldview) for its entities. While clinical drugs implicitly refer to a closed worldview, ingredients, clinical drug components and clinical drug forms can be understood in both open and closed worldview, leaving it to queries to distinguish between the two.

Finally, the Quantity Factor (QF) is a number followed by a unit of measure corresponding to vial sizes or patch durations (e.g., “12H”). RxNorm does not explicitly state whether strength is expressed as presentation strength or concentration strength. Presentation strength can be derived from concentration strength by multiplying the concentration strength by the quantity factor. (For example, if the concentration strength is 1MG/ML and the QF is 2ML, the presentation strength is 2MG/2ML). The Qualitative Distinction (QD) corresponds to some qualitative characteristic of a drug outside the main definitional features (e.g., “sugar free” and “abuse-deterrent”). QD and QF are optional modifiers used in RxNorm to define medicinal products when it is clinically relevant to identify such distinctions (12).

### Comparison of the RxNorm and SNOMED CT models

To compare the two models, we manually establish equivalences between their entities and between their definitional features, based on our analysis of the two models.

**Figure 3–:**
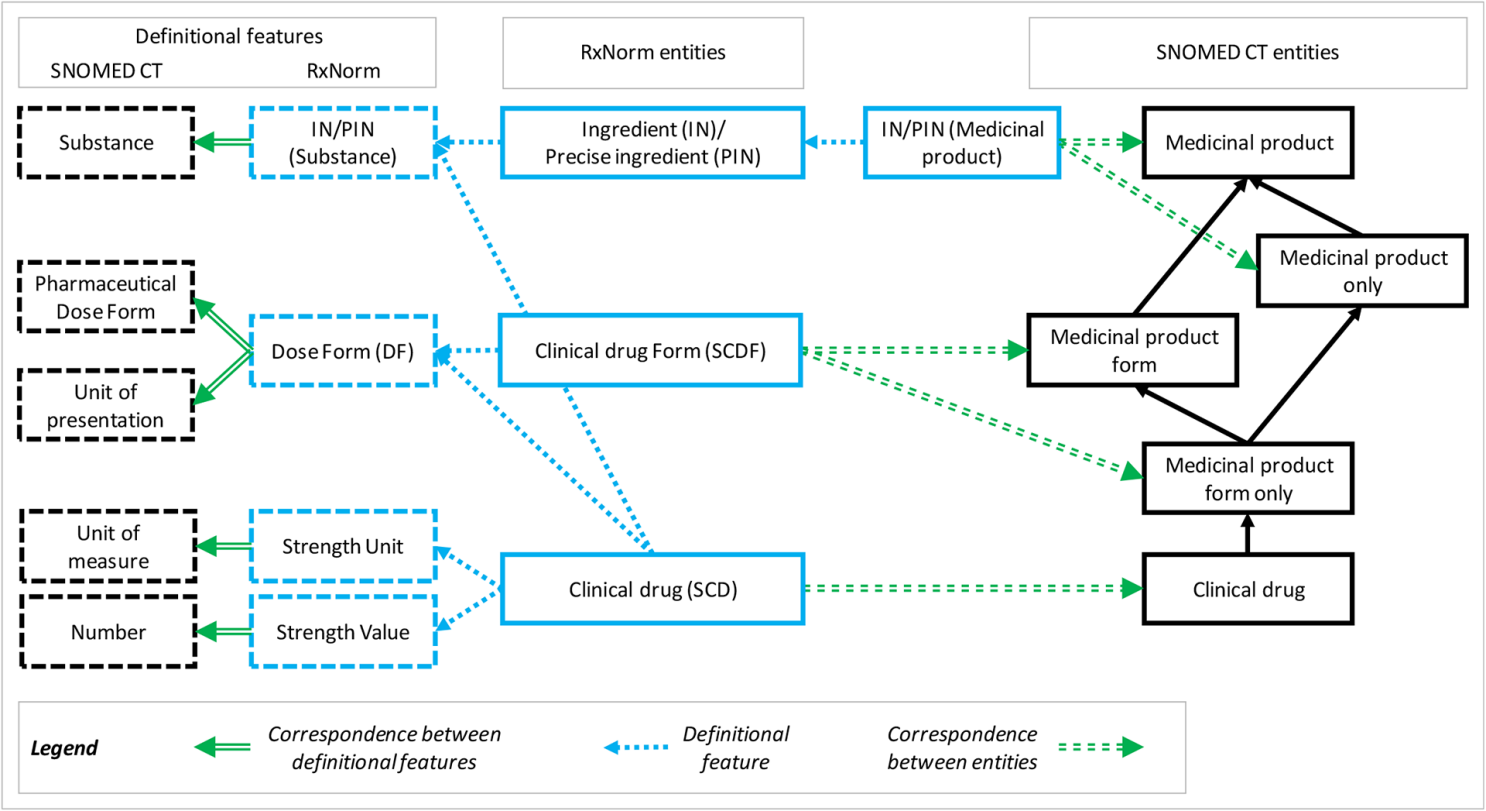
Correspondence between the RxNorm and SNOMED CT models

First, we need to disambiguate the notion of ingredient in RxNorm (IN,PIN, MIN), because, as mentioned earlier, it can be understood as either a substance or a class of medicinal products. Therefore, as shown in Figure 3, ingredients in RxNorm correspond to SNOMED CT medicinal products (in open and closed worldview) or to SNOMED CT substances, which are active ingredients of SNOMED CT medicinal products. In practice, RxNorm ingredients are often associated with multiple SNOMED CT entities, typically with one substance entity and one medicinal product entity. Disambiguation consists in identifying which SNOMED CT entity comes from the substance hierarchy (and treating it as a value for the definitional feature “active ingredient”), while the SNOMED CT entity corresponding to an entity from the medicinal product hierarchy is marked as an asserted equivalence for the RxNorm medicinal product entity.

RxNorm does not formally have the notion of “unit of presentation”. Units of presentation are implicitly represented through dose forms in RxNorm, whereas the two notions are represented separately in SNOMED CT. For example, in SNOMED CT, tablet is the logical “unit of presentation” of the conventional release oral tablet, while the two are conflated in the RxNorm dose form “Oral Tablet”. Therefore, RxNorm dose forms generally correspond to pairs of a pharmaceutical dose form and a unit of presentation in SNOMED CT.

In addition, there are no materialized entities for SCDCs in SNOMED CT. Instead, strength and basis of strength substance are associated as part of the definition of a clinical drug in SNOMED CT. Therefore, SCDCs cannot be related to entities in SNOMED CT, but their defining features are represented as part of clinical drug entities.

SCDs in RxNorm are equivalent to clinical drugs in SNOMED CT as they essentially share the same definitional features. The quantity factor in RxNorm has no direct equivalent in SNOMED CT, but QF information is implicitly represented in the presentation strength. In contrast, qualitative distinctions are absent from the SNOMED CT model.

While RxNorm only represents one level of modification (between PIN and IN), SNOMED CT can represent arbitrary levels of modification among substances.

Both RxNorm and SNOMED CT have the notion of concentration strength and presentation strength. However, RxNorm emphasizes concentration strength (from which presentation strength can be calculated using the quantity factor), whereas SNOMED CT explicitly represent both presentation strength and concentration strength when necessary.

Finally, RxNorm normalizes all quantities to one unit (per type of quantity), whereas SNOMED CT uses units that are most clinically appropriate (following IDMP requirements). For example, RxNorm uses 0.001 milligram and SNOMED CT 1 microgram. This difference merely reflects differences in editorial guidelines, as conversion between the two is trivial.

## Discussion

### Findings.

Not surprisingly, the models used by RxNorm and SNOMED CT for representing medicinal products are fairly similar and essentially compatible. Both models share major definitional features including ingredient (or substance), strength and dose form. Only the qualitative distinction feature of RxNorm has no correspondence at all in SNOMED CT.

SNOMED CT is more rigorous and better aligned with international standards. In SNOMED CT, differences tend to be made explicit, e.g., between a substance and the class of medicinal products containing this substance as an ingredient, or between the class of all medicinal products containing only a given active ingredient and the class of all medicinal products containing at least this active ingredient . SNOMED CT also offers more flexibility with relations among substances, as opposed to a fixed precise ingredient to base ingredient relationship in RxNorm. This precision comes at the price of a more complex model, and possibly a steeper learning curve. In contrast, RxNorm contains implicit knowledge, simplifications and ambiguities, but its model is simpler.

With features, such as explicit closed worldview for clinical drug entities, use of standard dose forms from EDQM, use of UCUM units, and use of clinically appropriate strength values, SNOMED CT shows better compliance with international standards (namely IDMP) than RxNorm does.

### Consequences on alignment.

Since their models are largely compatible, medicinal products from RxNorm and SNOMED CT are expected to be interoperable. However, specific aspects of the alignment between the two models require particular attention.

The values of **ingredient** can be aligned rather trivially (after disambiguation between the two meanings of RxNorm ingredients, substance and class of medicinal products containing this substance as an ingredient).

**Strength** entities require minimal attention, specifically for converting RxNorm “fixed unit” into the clinically appropriate unit used in SNOMED CT. Simple arithmetic is also required to convert concentration strength and quantity factor in RxNorm to presentation strength in SNOMED CT wherever appropriate.

In contrast, aligning **dose forms** requires more analysis, as RxNorm dose forms generally correspond to pairs of a pharmaceutical dose form and a unit of presentation in SNOMED CT.

The absence of correspondence for **qualitative distinction** in SNOMED CT may lead to multiple clinical drugs in RxNorm mapping to a single clinical drug in SNOMED CT. For example, the distinction between *Cholestyramine Resin 4000 MG Powder for Oral Suspension [RxCUI = 848943]* and its sugar-free form *Sugar-Free Cholestyramine Resin 4000 MG Powder for Oral Suspension [RxCUI = 1801279]* in RxNorm is lost in SNOMED CT. This issue is unlikely to result in clinically significant alignment errors.

The absence of materialization of the clinical drug component (SCDC) entity in SNOMED CT does not create an alignment issue, because SCDCs are essentially navigational entities in RxNorm. They are not crucial to any of the main use cases for RxNorm or SNOMED CT.

### Future work.

In future work, we plan to translate RxNorm into the SNOMED CT model for medicinal products. The resulting alignment would make RxNorm entities directly compatible with SNOMED CT’s. One benefit of this alignment would be to assess interoperability between RxNorm and SNOMED CT, potentially enriching SNOMED CT with clinical drugs currently specific to RxNorm. Additionally, this alignment would offer an opportunity for quality assurance by identifying cases where alignment is expected, but cannot be inferred (e.g., because of a difference in basis of strength substance for a given clinical drug between RxNorm and SNOMED CT).

## Conclusion

In this investigation, we examined the similarities and differences between the representation of medicinal products in RxNorm and SNOMED CT. We established that both models share major definitional features including ingredient (or substance), strength and dose form. Because of subtle differences between the two models, specific aspects of their alignment require particular attention.
